# Neonatal-onset familial hemophagocytic lymphohistiocytosis type 2: a case report

**DOI:** 10.3389/fped.2026.1965536

**Published:** 2026-09-25

**Authors:** Dongju Pan, Xueyu Li, Yushan Zhang, Shanping Chen, Jun Wu, Shuangyu Yang

**Affiliations:** Department of Pediatrics, Pu'er People’s Hospital, Pu’er, Yunnan, China

**Keywords:** familial hemophagocytic lymphohistiocytosis, genetic disease, genetic testing, neonate, PRF1 gene

## Abstract

**Background:**

Familial hemophagocytic lymphohistiocytosis type 2 (FHL2) is a rare autosomal recessive disorder primarily caused by mutations in the perforin-1 (PRF1) gene, leading to immune system hyperactivation and multi-organ dysfunction. FHL2 typically presents in infancy, with neonatal onset being extremely rare and particularly difficult to recognize early due to substantial clinical overlap with sepsis. Given its rapid progression, early detection is critical for effective diagnosis and management.

**Case summary:**

This case report describes a neonate with FHL2 who presented on day 8 after birth with fever (peak 38.9 °C), abdominal distension, and dyspnea, accompanied by coagulation dysfunction, liver function abnormalities, and capillary leak syndrome. Despite intensive care, the patient's condition continued to deteriorate, with progression to disseminated intravascular coagulation (DIC), polyserositis, and multi-organ failure. Despite receiving intensive care and comprehensive treatment, the patient's condition continued to deteriorate. By day 7 of hospitalization, the patient was in a moribund state, and the family requested discharge against medical advice; the patient died after discharge. Postmortem genetic testing identified a homozygous c.145G > A mutation in the PRF1 gene, resulting in a substitution of aspartic acid to asparagine at position 49 of the encoded protein (p.Asp49Asn). This mutation has been documented as pathogenic in the Human Gene Mutation Database.

**Conclusion:**

FHL2 may present in the neonatal period with complex and variable clinical manifestations, frequently leading to misdiagnosis as neonatal sepsis or DIC. When neonates present with unexplained persistent fever, cytopenia, coagulation dysfunction, and multi-organ dysfunction that fails to respond to conventional anti-infective therapy, HLH should be included in the differential diagnosis, and genetic testing should be performed promptly.

## 1 Introduction

Hemophagocytic lymphohistiocytosis (HLH) is a severe inflammatory syndrome mediated by immune dysregulation, characterized by excessive activation of lymphocytes and macrophages with massive release of inflammatory cytokines, potentially leading to multi-organ failure and death. HLH can be classified into primary (familial) and secondary types. Familial HLH (FHL) is an autosomal recessive disorder that typically presents in infancy and carries a poor prognosis (1).

The pathogenesis of FHL centers on impaired cytotoxic lymphocyte function. Under normal conditions, cytotoxic T lymphocytes (CTLs) and natural killer (NK) cells eliminate infected or abnormally activated antigen-presenting cells and macrophages via secretory granules containing perforin and granzymes, thereby terminating immune responses. When mutations occur in genes encoding critical proteins such as perforin (PRF1), Munc13-4 (UNC13D), syntaxin 11 (STX11), or Munc18-2 (STXBP2), the exocytosis of cytotoxic granules or perforin-mediated target cell lysis is impaired, resulting in ineffective clearance of antigens, persistent immune activation, and ultimately a fatal “cytokine storm” (2, 3). Several inborn errors of immunity (IEIs) associated with HLH have been identified. In addition to PRF1, UNC13D, STX11, and STXBP2, these include mutations in SH2D1A (XLP-1), XIAP, NLRC4, ITK, and CD27, all of which can manifest as HLH-like hyperinflammatory syndromes (4).

The diagnosis of HLH is based on the HLH-2004 diagnostic criteria established by the Histiocyte Society, requiring at least five of the following eight criteria to be met: fever, splenomegaly, cytopenia (affecting at least two lineages), hypertriglyceridemia and/or hypofibrinogenemia, elevated serum ferritin, decreased or absent NK cell activity, elevated soluble CD25 (sIL-2R), and hemophagocytosis detected in bone marrow or tissues (5). Furthermore, a definite family history or known genetic mutation is sufficient to diagnose familial HLH even when fewer than five of the above criteria are fulfilled (5). Standard treatment for HLH includes the HLH-2004 induction regimen (combination of dexamethasone, etoposide, and cyclosporine A) for rapid control of hyperinflammation, while hematopoietic stem cell transplantation (HSCT) remains the only curative approach for familial HLH, and early HSCT significantly improves long-term survival (6, 7).

Neonatal-onset HLH is extremely rare but poses substantial diagnostic challenges because the neonatal immune system is immature and clinical manifestations are often atypical. The presenting symptoms of neonatal HLH—fever, respiratory distress, abdominal distension, hepatosplenomegaly, and coagulation abnormalities—substantially overlap with common neonatal critical conditions such as sepsis and DIC, making misdiagnosis highly likely (8). Recently reported neonatal FHL2 cases similarly presented with fever, abdominal distension, hepatosplenomegaly, and coagulation dysfunction, were frequently misdiagnosed as sepsis, and were confirmed predominantly by postmortem genetic testing (9). Therefore, when neonates fail to respond to conventional anti-infective therapy and show rapid clinical deterioration, HLH should be included in the differential diagnosis, and genetic testing should be performed promptly.

This case report describes a neonate with FHL2, aiming to enhance clinicians’ awareness of this condition presenting in the neonatal period and to discuss its clinical features, diagnostic approach, and therapeutic challenges.

## 2 Case report

### 2.1 Basic information and clinical history

The patient was a female neonate, the first child of non-consanguineous Burmese parents. She was delivered by cesarean section at 39 + 1 weeks of gestation due to oligohydramnios at an outside hospital, with a birth weight of 2,950 g. The birth history was otherwise unremarkable. Both parents were healthy, with no family history of similar conditions.

On day 8 after birth, the infant developed fever (peak 38.9 °C), accompanied by abdominal distension and dyspnea. Laboratory tests at the outside hospital revealed thrombocytopenia (63 × 10⁹/L), and chest x-ray suggested bilateral multifocal pneumonia. The patient received penicillin, meropenem, oxygen therapy, and phototherapy for jaundice, but showed no improvement. Two days later, the patient was transferred to our neonatal intensive care unit due to clinical deterioration.

### 2.2 Physical examination at admission

On admission, the patient's vital signs showed a temperature of 38.3 °C, a heart rate of 195 beats/min, a respiratory rate of 65 breaths/min, a blood pressure of 72/44 mmHg, and a transcutaneous oxygen saturation of 86% on room air. Physical examination revealed generalized skin desquamation without maculopapular rash, along with mild jaundice, and the anterior fontanel was flat. Respiratory examination demonstrated tachypnea with coarse breath sounds bilaterally. The abdomen was distended with abdominal wall tension; the liver and spleen were not palpable below the costal margin, and bowel sounds were reduced to 2–3 times/min. The extremities were warm, and muscle tone was normal.

### 2.3 Laboratory and ancillary investigations

Laboratory findings on admission are summarized in Table 1.

**Table 1:** Laboratory findings on admission (on the day of admission).

Parameter	Result	Reference range
White blood cell count (WBC)	6.81 × 10⁹/L	4.3–14.2 × 10⁹/L
Neutrophil percentage	36.5%	7.0–56.0%
Lymphocyte percentage	48.1%	26.0–83.0%
Monocyte percentage	14.9%	3.0–16.0%
Eosinophil percentage	0.3%↓	1.0–10.0%
Basophil percentage	0.2%	0.0–1.0%
Platelet count	17 × 10⁹/L ↓	183–614 × 10⁹/L
Hemoglobin	92 g/L ↓	97–183 g/L
Fibrinogen	0.87 g/L ↓	2–4 g/L
Prothrombin time	17.90 s ↑	10–14 s
Activated partial thromboplastin time	71.50 s ↑	22.3–32.5 s
Thrombin time	30.10 s ↑	14–21 s
D-dimer	9.89 μg/mL ↑	0–0.55 μg/mL
Lactate dehydrogenase (LDH)	1,210 U/L ↑	120–250 U/L
Aspartate aminotransferase (AST)	294 U/L ↑	21–80 U/L
Alanine aminotransferase (ALT)	94 U/L ↑	8–71 U/L
*γ*-Glutamyl transferase (GGT)	360 U/L ↑	9–150 U/L
Total bilirubin	101.1 μmol/L ↑	0–21 μmol/L
Direct bilirubin	72.9 μmol/L ↑	0–10 μmol/L
Albumin	27.9 g/L ↓	35–50 g/L
C-reactive protein (CRP)	69.06 mg/L ↑	0–10 mg/L
Procalcitonin (PCT)	1.540 ng/mL ↑	<0.046 ng/mL
Interleukin-6 (IL-6)	176.7 pg/mL ↑	<7.0 pg/mL

Infection screening: Blood cultures identified ESBL-producing *Escherichia coli* and *Enterococcus faecium*; respiratory metagenomic next-generation sequencing (mNGS) identified *Staphylococcus aureus*, *Pseudomonas aeruginosa*, and *Acinetobacter baumannii*. TORCH IgM antibodies were all negative, with IgG positivity (maternal origin), excluding congenital TORCH infection. Hepatitis B, hepatitis C, syphilis, HIV, and hepatitis A IgM were all negative; hepatitis E IgG was positive (3.97 S/CO, representing maternal passive antibody). Cerebrospinal fluid (CSF) routine, biochemistry, and culture were normal, excluding purulent meningitis.

Imaging findings: Chest CT (Figure 1) showed patchy consolidation and ground-glass opacity in the right lower lobe, with a small right-sided pleural effusion and a small abdominopelvic effusion; no evidence of gastrointestinal perforation, intussusception, or obstruction was observed. Abdominal ultrasound revealed a small right-sided pleural effusion (depth 1.5 cm) and a small abdominopelvic effusion (depth 2.1 cm), with normal liver, gallbladder, pancreas, spleen, and kidneys. Follow-up abdominal ultrasound (Figure 2) demonstrated hepatomegaly with coarsened parenchymal echogenicity (suggestive of hepatic injury), thickening of the gallbladder wall with biliary sludge, mild splenomegaly, bilateral pleural effusion (right 1.7 cm, left 1.4 cm), and moderate abdominopelvic effusion (3.1 cm). Echocardiography revealed a patent foramen ovale (2 mm shunt) with normal cardiac function (EF 72%). Cranial ultrasound showed no intracranial hemorrhage or structural abnormalities. Hip ultrasound revealed bilateral Graf Type Ib hips (mild developmental immaturity).

**Figure 1:**
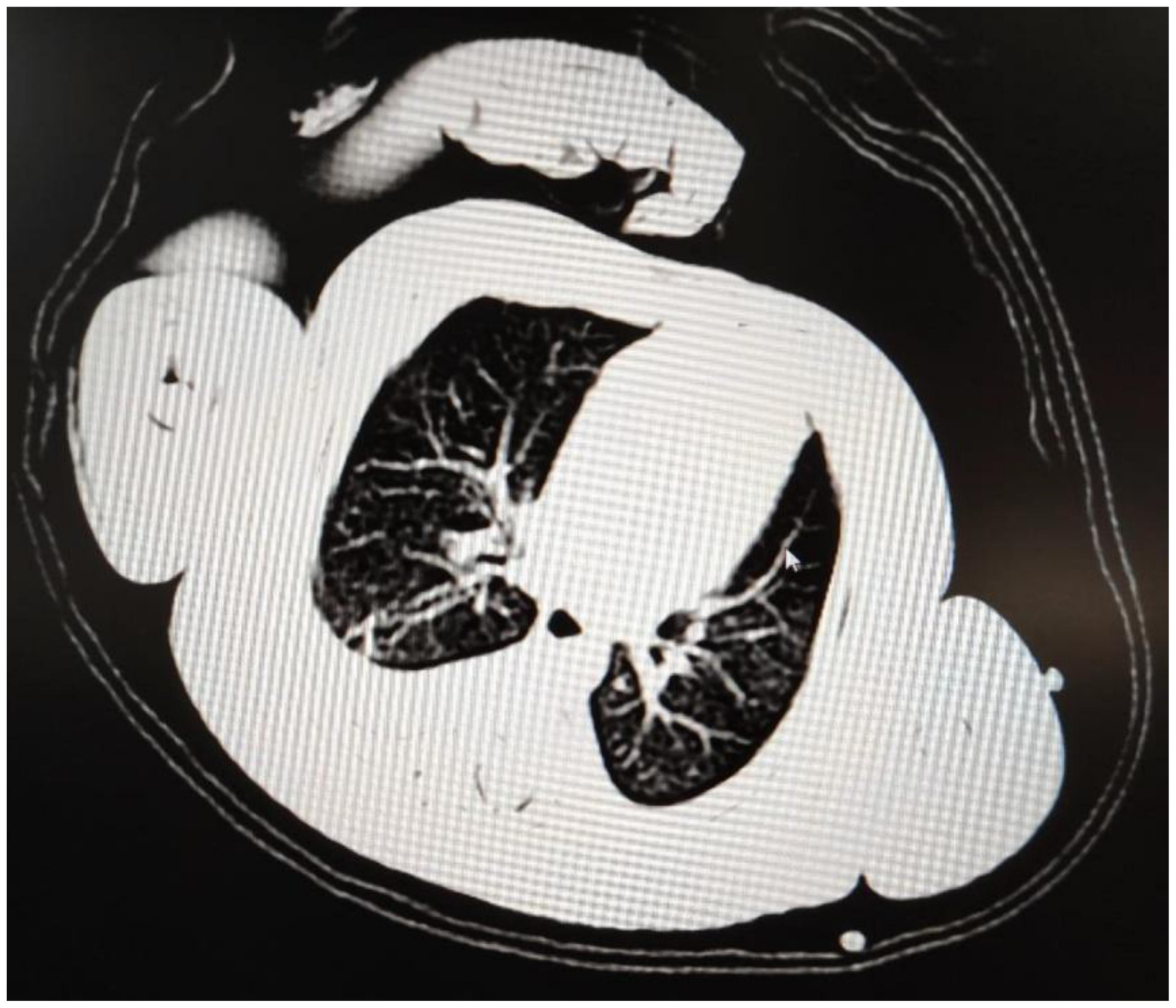
Chest CT. The CT scan shows patchy consolidation and ground-glass opacity in the right lower lung, suggestive of pneumonia.

**Figure 2:**
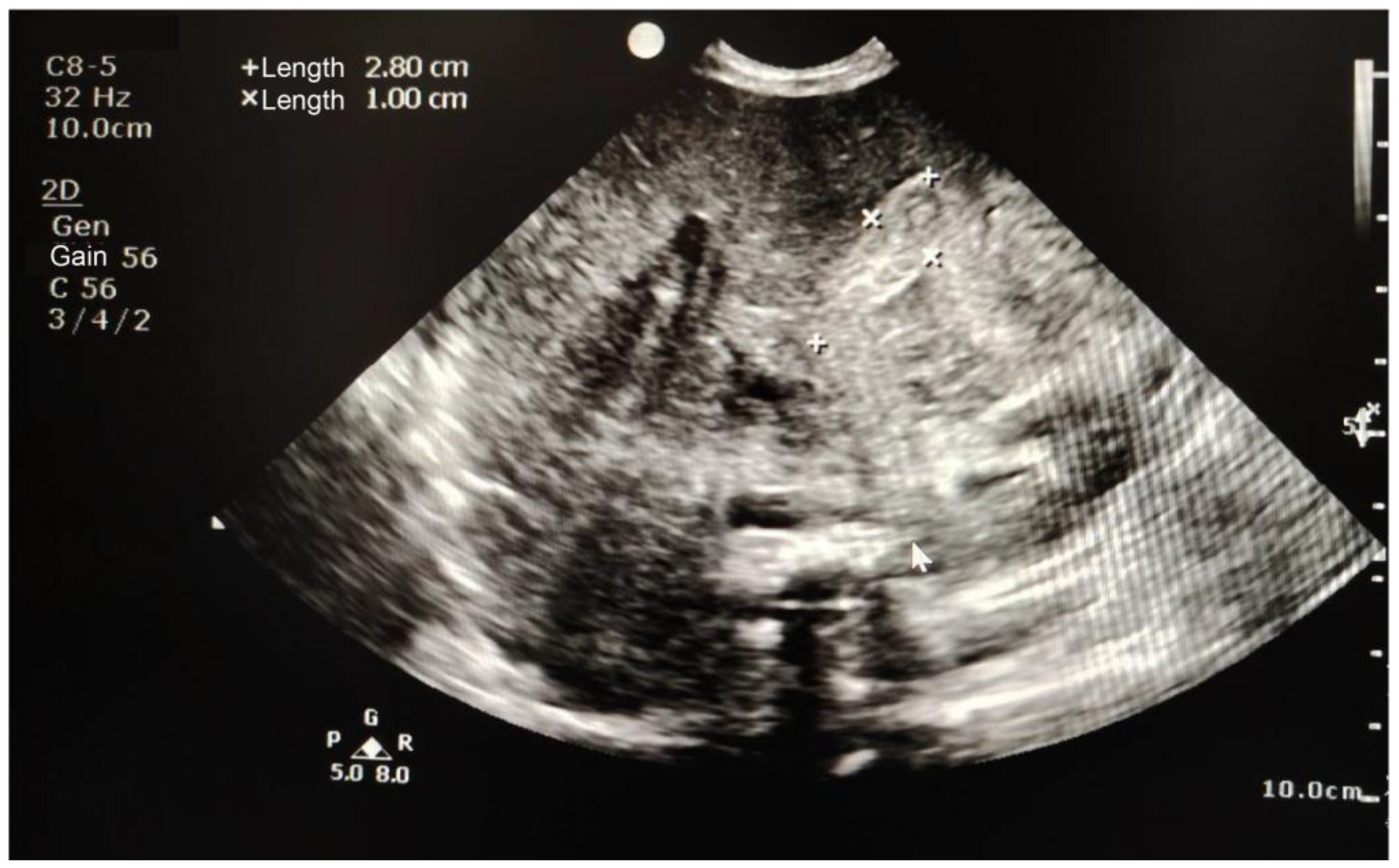
Abdominal ultrasound. Abdominal ultrasound showed hepatomegaly, slightly increased liver parenchymal echogenicity (suggestive of liver damage), thickening of the gallbladder wall, and abnormal echoes in the gallbladder (possibly bile sludge). Mild splenomegaly and bilateral pleural effusion were noted.

### 2.4 Therapeutic management and outcome

Upon admission, the infant received immediate intensive care, including fasting, gastric decompression, vitamin K1 for hemorrhage prevention, and piperacillin-tazobactam for infection.

During the initial phase of admission (days 1–2), due to a drop in platelets to 17 × 10⁹/L and fibrinogen to 0.87 g/L, DIC was suspected. Enoxaparin was administered subcutaneously for anticoagulation, and 60 mL of cryoprecipitate was transfused to correct coagulation dysfunction. Antibiotic therapy was escalated to meropenem combined with vancomycin. On day 2, respiratory distress worsened, requiring endotracheal intubation and invasive mechanical ventilation (SIMV mode). Because the patient showed poor response to conventional therapy, in addition to neonatal sepsis and DIC, a genetic metabolic disorder (including organic acidemias, fatty acid oxidation disorders, and mitochondrial diseases) was also strongly suspected in the differential diagnosis. Therefore, on day 5 of admission, with family consent, a blood sample was sent for genetic testing for rare neonatal disorders. Lumbar puncture on the same day was normal, excluding central nervous system infection. Methylprednisolone sodium succinate (2 mg/kg/day) was added for anti-inflammatory therapy, and azithromycin was administered for atypical pathogen coverage.

On days 3–5 of admission, the patient developed capillary leak syndrome (generalized pitting edema and progressive abdominal distension), gastrointestinal bleeding, and progressive hepatosplenomegaly (liver palpable 4 cm below the costal margin). Infectious parameters continued to deteriorate. On day 5, the patient's condition deteriorated precipitously. Follow-up abdominal ultrasound showed hepatomegaly with coarsened echogenicity (hepatic injury), gallbladder wall thickening with biliary sludge, mild splenomegaly, bilateral pleural effusion (right 1.7 cm, left 1.4 cm), and moderate abdominopelvic effusion (3.1 cm). Coagulation failure was evident (APTT >170 s, PT >170 s, fibrinogen 0.80 g/L), with platelets 20 × 10⁹/L, leukocytosis to 59.13 × 10⁹/L, ALT 1,352 U/L, AST 1,099 U/L, LDH >1,800 U/L, total bilirubin 282.5 μmol/L, direct bilirubin 207.1 μmol/L, and potassium 6.98 mmol/L. Despite multiple transfusions of cryoprecipitate, fresh frozen plasma, leukocyte-depleted red blood cells, and platelet apheresis, persistent multi-site bleeding developed. Due to refractory hypotension, continuous infusions of dopamine, dobutamine, and norepinephrine were initiated.

In the early morning of day 7 of admission (postnatal day 15): Oxygen saturation repeatedly dropped to 75%–80% and oliguria developed. Continuous renal replacement therapy (CRRT) was indicated but unavailable at our facility. After full discussion and disclosure of the grave prognosis, the family requested discharge against medical advice. The patient died shortly after discharge.

Eighteen days postmortem: Genetic testing revealed a homozygous c.145G > A mutation in the PRF1 gene (p.Asp49Asn). This mutation has been documented as pathogenic in the Human Gene Mutation Database (10). Genetic testing was performed using clinical exome sequencing and confirmed by Sanger sequencing. Based on the clinical presentation and genetic findings, the final diagnosis of FHL2 (OMIM:603553) was confirmed. The antemortem clinical diagnosis was revised to neonatal FHL2 (genetically confirmed postmortem) with multidrug-resistant bacterial sepsis. The clinical timeline is summarized in Table 2.

**Table 2:** Clinical timeline.

Time point	Major clinical events	Key laboratory findings	Therapeutic interventions
Day 8 after birth	Fever (38.9 °C), abdominal distension, dyspnea	Platelets 63 × 10⁹/L	Penicillin+meropenem (outside hospital)
Day of admission	Temperature 38.3 °C, RR 65/min, SpO₂ 86%	PLT 17 × 10⁹/L, FIB 0.87 g/L, LDH 1,210 U/L, CRP 69.06 mg/L	Piperacillin-tazobactam → meropenem+vancomycin; cryoprecipitate 60 mL
Day 2 of admission	Worsening respiratory distress, intubation	PLT 9 × 10⁹/L, Hb 69 g/L, FIB 0.95 g/L	Mechanical ventilation; RBC 65 mL; methylprednisolone, (2 mg/kg/d); lumbar puncture (normal)
Days 3–4 of admission	Generalized pitting edema, GI bleeding, hepatosplenomegaly	AST 391 U/L, ALT 99 U/L, DBil 55.7 μmol/L	Diuretics; albumin; glutathione; mNGS confirmed mixed infection
Day 5 of admission	Multi-site bleeding, coagulation failure, circulatory instability	PLT 20 × 10⁹/L, WBC 59.13 × 10⁹/L, ALT 1,352 U/L, K⁺ 6.98 mmol/L	Cryoprecipitate ×2, plasma 80 mL, RBC 65 mL, platelets 80 mL; norepinephrine; genetic testing sent
Day 7 of admission	SpO₂ 75%–80%, oliguria, family-requested discharge, and the patient died after discharge	—	Ventilator settings escalated; family requested discharge

## 3 Discussion

FHL2 (OMIM: 603553) is an autosomal recessive disorder caused by mutations in the PRF1 gene, resulting in perforin deficiency that impairs the ability of CTLs and NK cells to effectively eliminate activated macrophages, thereby leading to excessive inflammatory responses and multi-organ damage (11, 12). Neonatal-onset FHL is extremely rare and particularly difficult to recognize early, as its clinical manifestations—fever, respiratory distress, abdominal distension, hepatosplenomegaly, and coagulation dysfunction—substantially overlap with common neonatal critical conditions such as sepsis and DIC (10). This case precisely illustrates the great difficulty of identifying concomitant primary HLH in a critically ill neonate with proven infection: the patient had documented polymicrobial infection with multidrug-resistant organisms, severe DIC, and extremely rapid clinical deterioration, all of which severely limited the window for antemortem diagnosis. Therefore, the poor outcome should not be simply attributed to failure to recognize HLH earlier; rather, it should be recognized that the diagnostic challenge in such cases arises from the complex overlap between infection and primary HLH.

Postmortem genetic testing identified a homozygous c.145G > A mutation in the PRF1 gene, confirming the diagnosis of FHL2. Genotype–phenotype studies have shown that nonsense mutations in the PRF1 gene are significantly associated with earlier age of onset (*p* < 0.001) and complete absence of NK cell activity (13). Previously reported neonatal FHL2 cases similarly exhibit a hyperacute course, with death occurring within days to weeks after birth, and diagnosis is often confirmed by postmortem genetic testing (6, 14). Compared with previously reported cases, the unique feature of this case is the clear documentation of polymicrobial infection with multidrug-resistant organisms (ESBL-producing *Escherichia coli* and *Enterococcus faecium* on blood culture; *Staphylococcus aureus*, *Pseudomonas aeruginosa*, and *Acinetobacter baumannii* on respiratory mNGS), which further highlights the complexity of coexisting infection and FHL. This underscores the extremely fulminant clinical course of neonatal FHL2 and its exceedingly narrow therapeutic window.

According to the HLH-2004 diagnostic criteria (5), at least five of the following eight criteria must be met for a diagnosis of HLH (Table 3):

**Table 3:** HLH-2004 diagnostic criteria.

HLH-2004 criteria	Findings in this case	Met
1. Fever	Peak 38.9 °C, persistent fever	✅
2. Splenomegaly	Ultrasound showed mild splenomegaly	✅
3. Cytopenia (≥2 lineages)	Platelets lowest 9 × 10⁹/L, hemoglobin lowest 68 g/L	✅
4. Hypertriglyceridemia and/or hypofibrinogenemia	Fibrinogen lowest 0.80 g/L	✅
5. Ferritin ≥500 μg/L	Not tested	□
6. Decreased or absent NK cell activity	Not tested	□
7. Elevated sCD25 (sIL-2R)	Not tested	□
8. Hemophagocytosis in bone marrow/tissues	Bone marrow aspiration not performed due to coagulopathy	□

This case fulfilled criteria 1, 2, 3, and 4 (4 items). The remaining four items could not be completed for the following reasons: (1) ferritin, triglycerides, and sCD25 were not tested (retrospective case limitation); (2) NK cell activity and perforin expression were not tested (institutional limitations); and (3) bone marrow aspiration was contraindicated due to severe coagulopathy. Nevertheless, molecular evidence (homozygous pathogenic PRF1 mutation) independently establishes the diagnosis of familial HLH, independent of the completeness of the above laboratory investigations (5, 15).

Of note, even when some laboratory investigations are lacking, previously reported neonatal HLH cases often could not complete all HLH-2004 investigations due to the fulminant disease course, and diagnosis relied on postmortem genetic testing (6, 14). In the present case, hypofibrinogenemia, markedly elevated LDH (1,210 → > 1,800 U/L), and progressive thrombocytopenia all served as strong clues suggestive of HLH.

Regarding the differential diagnosis: Neonatal HLH and severe sepsis share extensive clinical overlap—both may present with fever, cytopenia, coagulation dysfunction, hepatosplenomegaly, and multi-organ involvement (8, 16). The infection screening results in this case revealed polymicrobial infection with multidrug-resistant organisms, which further complicated the differentiation from HLH, as infection itself can either trigger secondary HLH or serve as a trigger for FHL (17). In this case, the polymicrobial infection may have served as a trigger for FHL2, but the genetic basis of FHL2 remained the underlying etiology.

Regarding the exclusion of other inborn errors of immunity (IEIs): preliminary exclusion was performed through cerebrospinal fluid analysis, TORCH screening, viral marker testing, G6PD enzyme activity assay, and tandem mass spectrometry for metabolic disorders. However, normal routine immunological parameters are insufficient to completely exclude IEI. Due to the extremely rapid disease progression, whole-exome sequencing or targeted gene panels (covering SH2D1A, XIAP, NLRC4, ITK, CD27, and other HLH/IEI-related genes) could not be completed to comprehensively exclude other IEIs, which constitutes another important limitation of this case. Overall assessment indicates that the homozygous pathogenic PRF1 mutation is clearly established, and the clinical phenotype is highly consistent with FHL2, sufficient to establish the diagnosis of FHL2. Therefore, after genetic confirmation of FHL2, further whole-exome sequencing to exclude other IEIs was not pursued.

Regarding treatment: HLH-directed therapy (e.g., etoposide, cyclosporine A, emapalumab, or other immunomodulatory agents) was not initiated in this case for the following reasons: (1) the diagnosis of HLH was confirmed only 18 days postmortem through genetic testing and was not established during life; (2) the patient had a fulminant onset with rapid progression to DIC and circulatory failure, already being in the end-stage at the time genetic testing was sent; and (3) the patient's severe coagulopathy, shock, and multi-organ failure would have posed major challenges to HLH-directed therapy. However, whether earlier initiation of HLH-directed therapy would have altered the outcome in this case remains uncertain. This scenario suggests that for neonates in whom HLH is highly suspected, clinicians should not await the return of all HLH-2004 investigations or genetic testing results. Instead, while actively treating the infection, they should conduct parallel evaluation and management of infection and HLH/hyperinflammation, initiate empiric immunosuppressive therapy as early as possible, and urgently send genetic testing (6, 18). In the context of documented infection, infection should not be regarded as an absolute contraindication to initiating HLH therapy; rather, it should be recognized that infection can coexist with or serve as a trigger for primary HLH. The integrated interpretation of genetic and clinical findings is crucial for guiding early treatment decisions.

Clinical implications of this case: This case highlights the importance of early recognition and genetic testing in neonatal FHL2. Regarding genetic counseling and prenatal diagnosis: the patient carried a homozygous PRF1 mutation, indicating that both parents are heterozygous carriers. Due to the parents’ Burmese background and language barriers, parental PRF1 genetic testing could not be completed, which constitutes an important limitation of this case. FHL2 follows autosomal recessive inheritance, and when both parents are carriers, the recurrence risk for each subsequent pregnancy is 25% (5, 13). For families with a history of HLH or those who have previously had a child with FHL, prenatal genetic diagnosis, preimplantation genetic diagnosis (PGD), and neonatal early genetic screening are recommended in subsequent pregnancies. Previous literature has also emphasized the importance of genetic counseling and prenatal screening in high-risk families (19).

## Conclusion

This study reported a rare case of neonatal FHL2 and highlights the importance of early recognition and genetic testing. When neonates present with unexplained persistent fever, cytopenia, coagulation dysfunction, hepatosplenomegaly, and multi-organ failure that fails to respond to conventional anti-infective therapy, HLH should be included in the differential diagnosis. Because neonatal FHL2 progresses extremely rapidly, clinicians should not await the return of all diagnostic investigations but should initiate empiric immunosuppressive therapy and send urgent genetic testing as early as there is a high index of suspicion. Furthermore, genetic counseling and prenatal diagnosis should be strengthened in high-risk families to reduce the risk of recurrence of FHL2 in subsequent pregnancies in affected families and improve patient survival.

## Data Availability

The raw data supporting the conclusions of this article will be made available by the authors, without undue reservation.
